# microRNA-4331 Promotes Transmissible Gastroenteritis Virus (TGEV)-induced Mitochondrial Damage Via Targeting RB1, Upregulating Interleukin-1 Receptor Accessory Protein (IL1RAP), and Activating p38 MAPK Pathway In Vitro*[Fn FN2]

**DOI:** 10.1074/mcp.RA117.000432

**Published:** 2017-12-07

**Authors:** Xiaomin Zhao, Xiaoyuan Bai, Lijuan Guan, Juejun Li, Xiangjun Song, Xuelian Ma, Jianxiong Guo, Zhichao Zhang, Qian Du, Yong Huang, Dewen Tong

**Affiliations:** From the ‡College of Veterinary Medicine, Northwest A&F University, Yangling, Shaanxi 712100, P.R. China

## Abstract

Transmissible gastroenteritis virus (TGEV), a member of the coronaviridae family, could cause fatal diarrhea of piglets and result in numerous economic losses. Previous studies demonstrated that TGEV infection could lead to mitochondrial damage and upregulate miR-4331 level. So miR-4331 may play an important regulatory role in the control of mitochondrial function. To explore the potential role of miR-4331 in mitochondrial damage, we adopted a strategy consisting of quantitative proteomic analysis of porcine kidney (PK-15) cells in response to miR-4331 and TGEV infection. Eventually, 69 differentially expressed proteins were gained. The target of miR-4331 was identified. The effects of miR-4331 and its target RB1 on mitochondrial Ca^2+^ level, mitochondrial membrane potential (MMP), interleukin-1 receptor accessory protein (IL1RAP), p38 MAPK signaling pathway were investigated. The results showed that miR-4331 elevated mitochondrial Ca^2+^ level, reduced MMP, targets Retinoblastoma 1 (RB1), upregulated IL1RAP, and induced activation of p38 MAPK pathway during TGEV infection. RB1 was identified as the direct targets of miR-4331 and downregulated IL1RAP, suppressed the activation of p38 MPAK, and attenuated TGEV-induced mitochondrial damage. In addition, IL1RAP played a positive role in activating p38 MAPK signaling and negative role in TGEV-induced mitochondrial damage. The data indicate that miR-4331 aggravates TGEV-induced mitochondrial damage by repressing expression of RB1, promoting IL1RAP, and activating p38 MAPK pathway.

Transmissible gastroenteritis virus (TGEV)^1^ is an enveloped enteropathogenic coronavirus with positive-sense single-stranded RNA genome (1). TGEV infects pigs and especially causes piglets up to 14 days of age high mortality, which can reach to 100% (2, 3) and result in numerous economic losses. Our previous work demonstrated that TGEV infection can reduce MMP, leading to mitochondrial damage (4, 5). Mitochondrion, an organelle of eukaryocyte, not only acts as the energy metabolism factory, but also is involved in many key biological processes, such as apoptosis, pathogenic infection (6, 7). Mitochondrion is a very sensitive organelle to microenvironmental changes in cells and much easier becomes dysfunction than other organelles (8). Mitochondria, the major source of energy, in the form of ATP, are closely interconnected with cell death. Ca^2+^ is essential for maintain mitochondrial function. However, Ca^2+^ accumulation in mitochondria can impair mitochondrial function, resulting in a transient depolarization of MMP and reducing ATP production (9–11). Both Ca^2+^ accumulation and MMP depolarization can cause mitochondrial damage, so the alterations of Ca^2+^ level and MMP are used to assess mitochondrial function (12). miRNAs are small noncoding RNA species containing about 22 nucleotides and contribute to regulating many cellular processes including apoptosis, development, differentiation, and cell cycle (13). We previously reported that miR-4331 level was upregulated during TGEV-induced mitochondrial dysfunction (14). Whereas, whether miR-4331 participate in regulating TGEV-induced mitochondrial damage in PK-15 cells is unclear. According to GO enrichment and KEGG pathway analysis of miR-4331 targets, miR-4331 targets can be enriched in mitochondria and mitochondria-related pathways. Therefore, we postulate miR-4331 can participate in regulating mitochondrial dysfunction in PK-15 cells. To investigate the regulatory effects of miR-4331 on TGEV-induced mitochondrial damage, miR-4331 mimics or mimics control was transfected into PK-15 cells and the proteomic analysis was performed using LC-MS/MS labeled by Tandem Mass Tags (TMT). Then we identified the targets of miR-4331 and detected the effects of miR-4331 target on mitochondrial damage and p38 MAPK pathway. We demonstrated that miR-4331 increased mitochondrial Ca^2+^ level and decreased MMP via activating p38 MAPK pathway and upregulating expression of IL1RAP. Moreover, miR-4331 activated p38 MAPK pathway via targeting RB1 and upregulating IL1RAP. Our data suggest that miR-4331 could facilitate TGEV-induced mitochondrial damage in PK-15 cells via targeting RB1, promoting IL1RAP, and activating p38 pathway.

## EXPERIMENTAL PROCEDURES

### 

#### 

##### Antibodies and Plasmids

Anti-RB1 was purchased from Santa Cruz Biotechnology. Horseradish peroxidase (HRP)-conjugated secondary antibody was purchased from Pierce (US). Anti-p-p38 and anti-β-actin antibody were purchased from Cell Signaling Technology (US). Anti-IL1RAP and anti-GAPDH primary antibody was purchased from GeneTex (US). The psiCHECK-2 plasmid and dual-luciferase reporter assay system were purchased from Promega (US). The primers and siRNAs were synthesized by Ribo Biotech (RiboBio, China).

##### Virus and Cells

The TGEV Shaanxi strain was isolated from TGEV-infected piglets (15). PK-15 cells were obtained from ATCC (CCL-33) and grown in Dulbecco's Minimal Essential Medium (DMEM) supplemented with 10% fetal bovine serum (Hyclone, US), 100 IU of penicillin, and 100 mg/ml streptomycin, at 37 °C in 5% CO_2_ atmosphere incubator.

##### Experimental Design and Statistical Rationale for Proteomics

In order to investigating the effect of miR-4331 on mitochondrial damage in response to TGEV infection, PK-15 cells were transfected with 100 nm miR-4331 mimics or negative control using Lipofectamine 3000 and infected with TGEV at an MOI of 1.0 at 24 h post-transfection (hpt). The cells were collected for quantitative proteomic analysis at 24 h post-infection (hpi). Two biological replicates preparations labeled with TMT were analyzed. To evaluate the transfection efficiency, the miR-4331 level was measured by real-time PCR. Reverse transcription reactions and real-time PCR were performed as described previously (14). The relative quantification of miRNAs was normalized to U6 using the two-ddCt method (16).

##### Protein Isolation, Digestion, and Labeling with TMT

The PK-15 cells were sonicated on ice in lysis buffer (8 m urea, 1% Triton-100, 65 mm DTT and 0.1% protease inhibitor mixture) and centrifuged at 12,000 × *g* for 20 min at 4 °C. The clarified supernatant was collected. Finally, the protein was precipitated with cold 15% TCA for 2 h at −20 °C and centrifuged at 4 °C for 10 min. The precipitate was redissolved in buffer (8 m urea, 100 mm TEAB, pH 8.0). One hundred micrograms protein of each sample was digested with trypsin. The two control samples and two treatment samples were respectively labeled with TMT (126, 129, 127, and 130).

##### LC-MS/MS

The samples were fractionated by high pH reverse-phase High Performance Liquid Chromatography (HPLC). Briefly, peptides were dried by vacuum centrifugation. Then the Peptides were dissolved in 0.1% formic acid and directly loaded onto a reversed-phase precolumn (Acclaim PepMap 100, Thermo Scientific). Peptides were separated using a reversed-phase analytical column (Acclaim PepMap RSLC, Thermo Scientific) and analyzed by Q Exactive^TM^ hybrid quadrupole-Orbitrap mass spectrometer (Thermo Fisher Scientific).

The peptides were subjected to NSI source followed by tandem mass spectrometry (MS/MS) in Q Exactive^TM^ (Thermo Fisher Scientific) coupled online to the HPLC. Intact peptides were detected in the Orbitrap at a resolution of 70,000. Peptides were selected for MS/MS using NCE setting as 32. Ion fragments were detected in the Orbitrap at a resolution of 17,500. A data-dependent procedure that alternated between one MS scan followed by 20 MS/MS scans was applied for the top 20 precursor ions above a threshold ion count of 2E4 in the MS survey scan with 30.0 s dynamic exclusion. The electrospray voltage was 2.0 kV. Automatic gain control (AGC) was used to prevent overfilling of the ion trap. 5E4 ions were accumulated for generation of MS/MS spectra. For MS scans, the *m*/*z* scan range was 350 to 1800. Fixed first mass was set as 100 *m*/*z*.

##### Analysis of LC-MS/MS Data

MS/MS data were analyzed by Mascot (version 2.3.0) according to a previously described protocol (17). Briefly, raw MS data files were processed using the LC/MS software Proteome Discoverer (version 1.3.0.339) (Thermo scientific, US) and converted into the Mascot generic format (mgf) files. Mascot software performed peak generation, precursor mass recalibration, extraction of TMT 6-plex reporter ions intensity, and calculation of TMT 6plex reporter ions intensity ratios. For each MS/MS spectra, top 10 most intense peaks in every 100 Da window were extracted for database search. Then Mascot search was performed by searching tandem mass spectra against Uniprot Sus scrofa database (UniProt release 2015_04, 26054 entries) (http://www.uniprot.org) concatenated with reversed decoy database and protein sequences of common contaminants. Cleavage enzyme was specified as trypsin/P, maximum number of missing cleavages was set to 2. Mass tolerance for precursor ion was set to 10 ppm and mass tolerance for MS/MS ions was set to 0.02 Da. Carbamyiodomethylation on Cys and TMT6plex (N-term) and TMT6plex (K) were specified as fixed modification and oxidation on Met, acetylation on protein N-terminal were specified as variable modifications. False discovery rate (FDR) thresholds for protein, peptide and modification site were specified at 1%. Mascot software assembled the peptide/protein groups, calculated false discovery rates, filter the identifications. Protein quantification was set to use only unique peptides bearing any modification. The median ratio of TMT 6-plex reporter intensity of unique peptides was set as protein relative abundance changes. In this study, we prepared two duplicate samples. For each protein, we set the average ratio calculated from two duplicate samples as the final quantitation of protein. Student's test was explored to calculate differential significance degree of protein relative abundance changes. *t* test *p* value less than 0.05 was considered significantly differentially. The mass spectrometry proteomics data have been deposited to the ProteomeXchange Consortium via the PRIDE partner repository with the data set identifier PXD008174 (http://www.ebi.ac.uk/pride/archive/) (18).

##### Bioinformatic Analysis of Differentially Expressed Proteins

Gene Ontology (GO) annotation of proteome was derived from the UniProt-GOA database (http://www.ebi.ac.uk/GOA). Kyoto Encyclopedia of Genes and Genomes (KEGG) database was used to annotate proteins pathways. The Wolf Psort database (https://wolfpsort.hgc.jp/) was used to predict subcellular localization.

##### Analysis of Protein-Protein Interactions

The differentially expressed protein name identifiers (Uniprot accession) were searched against the STRING database (version 10.0) for protein-protein interactions (19).

##### Vector Construction

To overexpress RB1 and IL1RAP, the full-length sequences of RB1 and IL1RAP were obtained by PCR from PK-15 cells and cloned into plasmid pCI-neo (Promega, USA). The constructions were respectively named pCI-neo-RB1 and pCI-neo-IL1RAP. The primer sequences are shown in supplemental Information S1.

##### Western Blot Analysis

PK-15 cells were treated with RIPA lysis buffer containing phenylmethyl sulfonylfluoride (PMSF). The proteins were separated using 12% sodium dodecyl sulfate-polyacrylamide gel electrophoresis (SDS-PAGE) and transferred onto the polyvinylidene difluoride (PVDF) membrane. The membrane was blocked with 5% BSA for 2 h at room temperature and then incubated with primary antibodies overnight at 4 °C. The HRP-conjugated secondary antibodies were used to incubate the membrane for 1 h at room temperature.

##### Reverse Transcription PCR and Real-time PCR

Total RNA was extracted with TRIzol (Invitrogen, US). 2 μg of total RNA was treated with DNase I (Fermentas, Germany) for 30 min at 37 °C and reversely transcribed using the First-strand cDNA synthesis kit (Invitrogen). The cDNA was used as the template for real-time PCR. The real-time PCR was performed on Bio-Rad iQ5 Real-Time PCR System (Bio-Rad). The reverse transcription primers and real-time PCR primers are shown in supplemental Information S1.

##### Dual-luciferase Reporter Assay

3′ UTRs of candidate targets containing the miR-4331 binding sites were respectively amplified by PCR and were cloned into the vector psiCHECK-2 (Promega). The binding sites of miR-4331 in RB1-wt 3′ UTR were mutated following the mutagenesis protocol (20) to generate RB1-mut. The PCR primers are shown in supplemental Information S1. PK-15 cells were co-transfected with plasmid RB1-wt (or RB1-mut) and miR-4331 mimics (or miR-4331 inhibitors) using Lipofectamine 3000 (Invitrogen, US). The luciferase activities were detected at 48 hpt using a Dual-Glo Luciferase Assay System (Promega).

##### RNA Interference

The siRNAs of RB1 and IL1RAP were synthesized by GenePharma (GenePharma, China) (supplemental Information S1). PK-15 cells were transfected with 100 nm siRNA or irrelevant siRNA using Lipofectamine 3000 (Invitrogen, US).

##### Evaluation of MMP

MMP of PK-15 cells was detected using JC-1 as an indicator (21). PK-15 cells were transfected with miR-4331 mimics or miR-4331 inhibitors and were subsequently infected with TGEV at 1 MOI. PK-15 cells were treated with PBS containing JC-1 and incubated at 37 °C for 20 min. The absorbance was measured at 550 ex/590 em.

##### Measurement of Mitochondrial Ca^2+^ Level

The mitochondrial calcium level was detected using Rhod-2 kit (GENMED, China) following the manufacturer's protocol. The absorbance was measured at 490 ex/590 em. The relative fluorescence unit (RFU) was calculated.

## RESULTS

### 

#### 

##### TGEV Infection Increases Mitochondrial Ca^2+^ Level and Decreases MMP

**Fig. 1.**
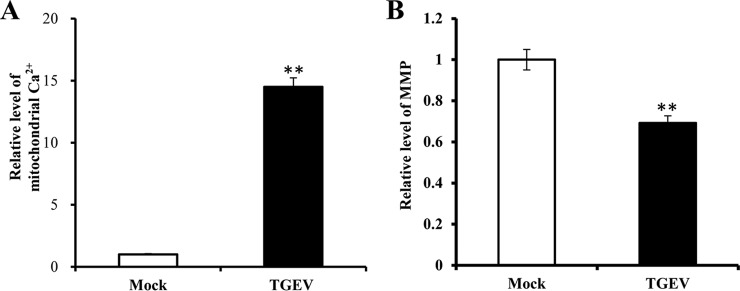
**The effects of TGEV infection on mitochondrial Ca^2+^ level and MMP.** *A,* Mitochondrial Ca^2+^ level in PK-15 cells; *B,* MMP of PK-15 cells. Data are representative three independent experiments. ** *p* < 0.01.

PK-15 cells were infected with TGEV at 1 MOI for 24 h. The mitochondrial Ca^2+^ level and MMP of PK-15 cells were evaluated. The results showed that TGEV infection increased mitochondrial Ca^2+^ level (Fig. 1*A*) and decreased mitochondrial MMP (Fig. 1*B*).

##### Gene Ontology Enrichment Analysis of miR-4331 Targets

**Fig. 2.**
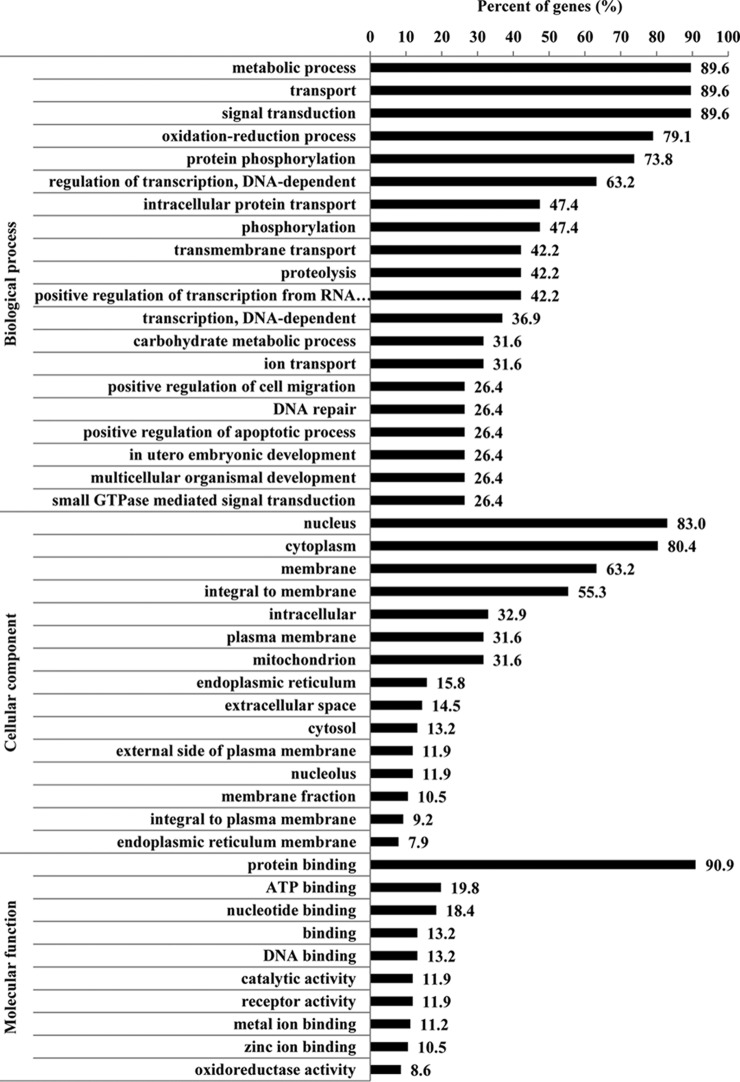
**GO enrichment analysis of miR-4331 targets.**

miR-4331 targets were predicted using TargetScan and miRanda, by which 618 targets were obtained (supplemental Information S2). The 618 targets of miR4331 were searched against Gene Ontology (GO) database to provide enrichment information on biological processes, molecular functions, and cellular components. Among total 618 predicted targets, 26 targets were related to mitochondria, 4.2% of total targets. GO enrichment of the 618 targets of miR-4331 showed that 89.6% of targets were enriched in metabolic process and 31.6% of targets were linked to mitochondria in cellular component (Fig. 2).

##### miR-4331 Increases Mitochondrial Ca^2+^ Level and Decreases MMP

**Fig. 3.**
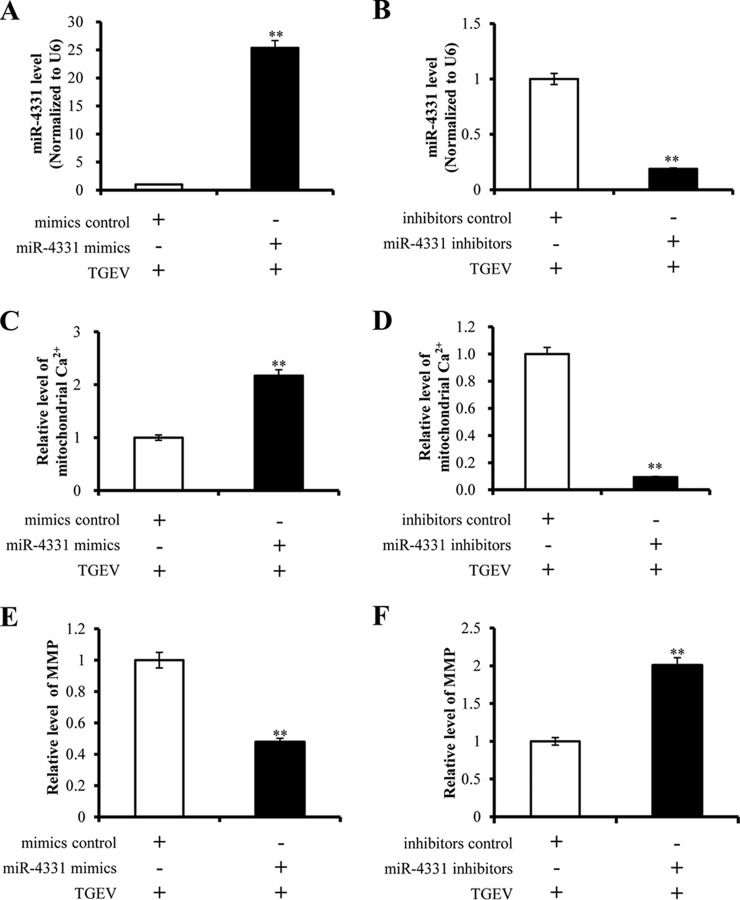
**The effects of miR-4331 on mitochondrial Ca^2+^ level and MMP in PK-15 cells infected with TGEV for 24 h.** *A,* miR-4331 level in PK-15 cells transfected with miR-4331 mimics (or miRNA mimics control) and infected with TGEV for 24 h. The miR-4331 level was measured using real-time PCR (normalized to U6 and in reference to the level of the control); *B,* miR-4331 level of PK-15 cells transfected with miR-4331 inhibitors (or miRNA inhibitors control) and infected with TGEV for 24 h The miR-4331 level was measured using real-time PCR (normalized to U6 and in reference to the level of the control); *C* and *D,* The effect of miR-4331 on mitochondrial Ca^2+^ level during TGEV infection. *E* and *F,* The effect of miR-4331 on MMP during TGEV infection. Data are representative three independent experiments. ** *p* < 0.01.

We previously reported that TGEV infection caused the reduction of MMP through inducing ROS accumulation and increased miR-4331 level (5, 14). To investigate the effects of miR-4331 on mitochondria damage during TGEV infection, the PK-15 cells were transfected with miR-4331 mimics, or miRNA mimics control, miR-4331 inhibitors, inhibitors, and subsequently infected with TGEV at 1 MOI for 24 h. The miR-4331 level remarkably increased by miR-4331 mimics (Fig. 3*A*) and suppressed by miR-4331 inhibitors (Fig. 3*B*). miR-4331 mimics led to a decrease of mitochondrial Ca^2+^ level (Fig. 3*C*) and an increase of MMP (Fig. 3*E*). miR-4331 inhibitors reduced mitochondrial Ca^2+^ level (Fig. 3*D*) and increased MMP (Fig. 3*F*).

##### Quantitative Proteomic Analysis

A total of 4209 proteins were identified by high-resolution LC-MS/MS analysis, among which 4165 proteins were quantified (supplemental Information S3). When setting ≥1.2-fold as the upregulated threshold and ≤0.83-fold as the downregulated threshold, 69 differentially expressed proteins were obtained, including 33 upregulated proteins and 36 downregulated proteins (Table I). Raw data of MS are available via ProteomeXchange with identifier PXD008174.

**Table I:** Differentially expressed proteins of PK-15 cells induced by miR-4331 during TGEV infection^a^

No.	Protein	Fold change	*p* value	No.	Protein	Fold change	*p* value
1	GBP1	1.636	2.86082E-08	36	LEMD3	0.827	0.033892233
2	SDC4	1.6075	0.010935485	37	TPP1	0.827	0.008198212
3	Uncharacterized protein	1.4665	0.003411649	38	PHLDB1	0.821	0.046323642
4	MRPL11	1.4465	0.023362276	39	L3MBTL3	0.8185	0.001753769
5	HK2	1.417	1.38772E-07	40	CDH6	0.818	0.004508034
6	LOC100522887	1.411	0.004014107	41	CAV1	0.818	8.42356E-06
7	LAMA3	1.382	0.014635659	42	MAGED2	0.817	0.015630659
8	HELLS	1.322	0.009530397	43	PTPN12	0.817	0.00278265
9	LOC100512420	1.3185	0.029473603	44	PDLIM5	0.817	0.009683894
10	LOC100516293	1.3175	0.011026956	45	DDX54	0.813	0.022637134
11	SLC7A2	1.312	0.005947393	46	SNX5	0.812	0.001798921
12	HMGCS1	1.311	0.000560657	47	AHNAK	0.805	2.69598E-10
13	P4HA1	1.303	0.002036451	48	G3BP1	0.799	0.003535946
14	NDUFA13	1.294	0.018110837	49	LOC100522554	0.795	0.001425858
15	IL1RAP	1.28	0.000180531	50	LOC100621379	0.79	0.003769048
16	TACSTD2	1.275	0.004011772	51	CPNE3	0.786	0.029391873
17	E-cadherin	1.272	0.004320702	52	CAMK2D	0.781	0.01838761
18	PLOD1	1.272	0.022204118	53	ERAP1	0.777	0.000161803
19	ITM2B	1.2665	0.011878292	54	DAB2	0.771	0.017019972
20	COX5A	1.262	0.017952372	55	MX1	0.767	7.97178E-09
21	HSPG2	1.256	0.001915952	56	SNX2	0.763	0.005488335
22	F11R	1.2465	0.018738586	57	PSAT1	0.755	0.038400715
23	TOP1	1.243	6.08874E-08	58	LOC100522926	0.755	0.00766312
24	NDUFB5	1.238	0.048158959	59	BRI3BP	0.7475	0.041541833
25	ANXA1	1.227	0.033945569	60	PCK2	0.739	0.02966721
26	NDUFS1	1.224	3.01379E-06	61	SRP72	0.7225	0.000330778
27	MRPL47	1.222	0.006827477	62	BCAT1	0.7205	0.00291414
28	RELA	1.219	0.002508153	63	Jup	0.696	0.015611879
29	CD47	1.2185	0.042185225	64	LOC100524144	0.6195	0.000260732
30	LOC100513756	1.214	0.02940679	65	RAPH1	0.614	0.018306286
31	MRPL24	1.214	0.023142371	66	ADFP	0.58	0.0030315
32	CYP51A1	1.213	0.002272823	67	CWF19L1	0.561	0.001892935
33	FN1	1.204	2.01222E-06	68	DSG2	0.504	0.000252131
34	STAT5a	0.8315	0.005207431	69	PGCP	0.388	0.045491304
35	TAP2	0.83	0.005291123				

^a^ More information is available in supplemental Information S4.

##### GO Enrichment Analysis of the 69 Differentially Expressed Proteins

**Fig. 4.**
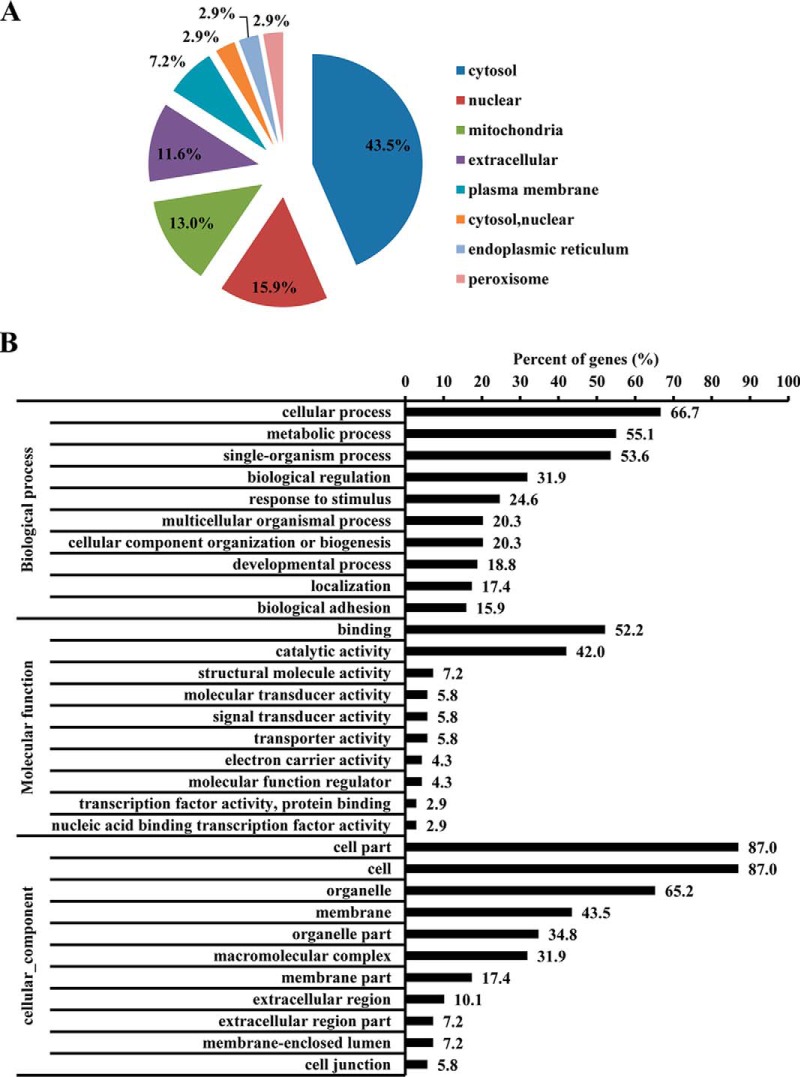
**Subcellular localization and GO analysis of differentially expressed proteins.** PK-15 cells were transfected with miR-4331 mimics (or miRNA mimics control) and infected with TGEV for 24 h. *A,* Subcellular localization of differentially expressed proteins; *B,* GO enrichment analysis of differentially expressed proteins.

The 69 differentially expressed proteins were searched against Wolf Psort database (https://wolfpsort.hgc.jp/) for prediction of subcellular localization. The subcellular localization analysis revealed that the distribution of differentially expressed proteins was distributed in cytosol (43.48%), nuclear (15.49%), mitochondria (13.04%), extracellular (11.59%), plasma membrane (7.25%), cytosol and nuclear (2.9%), endoplasmic reticulum (2.9%), and peroxisome (2.9%) (Fig. 4*A*). The 69 differentially expressed proteins were annotated by UniProt-GOA database or InterProScan. The proteins were enriched by GO annotation based on three categories: biological process, cellular component, and molecular function. The differentially expressed proteins are primarily enriched in cellular process (66.67%), metabolic process (55.07%), and single-organism process (53.62%). The molecular functions of the differentially expressed proteins are mainly involved in binding (52.17%) and catalytic activity (42.03%). In addition, the differentially expressed proteins are the component of cell part (86.96%), cell (86.96%), and organelle (65.22%) (Fig. 4*B*).

##### KEGG Pathway Enrichment Analysis of Differentially Expressed Proteins

**Fig. 5.**
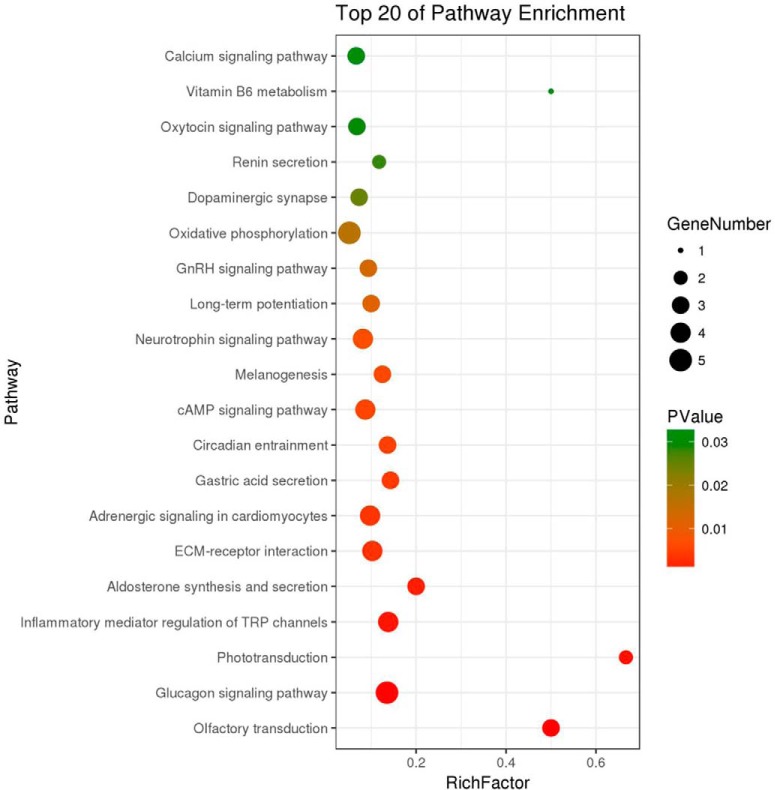
**KEGG pathway enrichment analysis of differentially expressed proteins.**

**Fig. 6.**
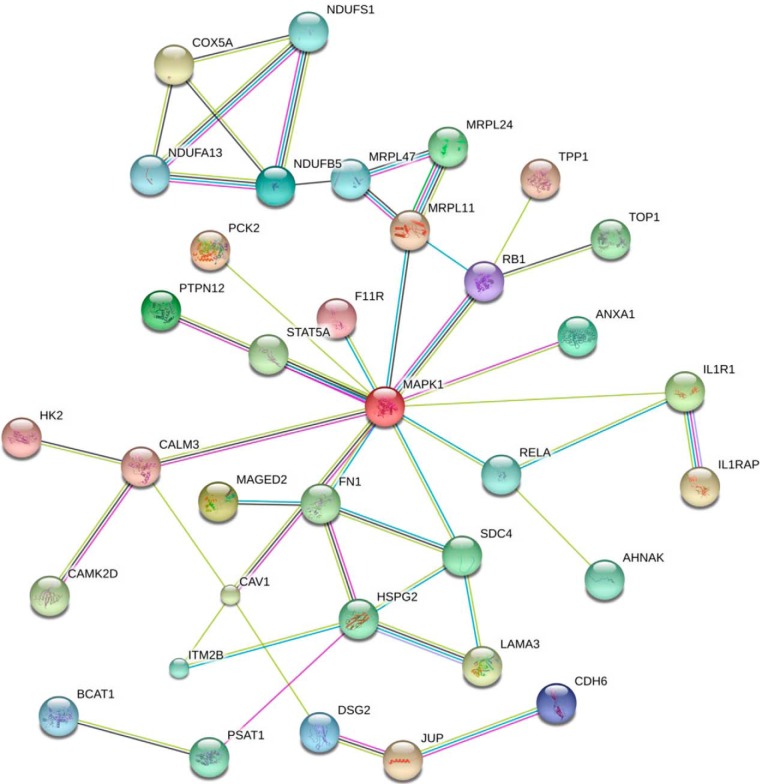
**Interactions of differentially expressed proteins.** Specific interaction networks analysis of differentially expressed proteins. Network nodes represent proteins. Splice isoforms or post-translational modifications are collapsed, *i.e.* each node represents the protein produced by a single, protein-coding gene locus. Colored nodes represent query proteins and the first shell of interactors. White nodes represent the second shell of interactions. Small nodes are the proteins with unknown 3D structure. Large nodes are the proteins with known or predicted 3D structure. Lines represent protein-protein interactions. Known Interactions: 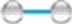 represents from curated databases; 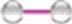 represents experimentally determined. Predicted Interactions: 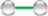 represents gene neighborhood; 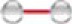 represents gene fusions; 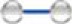 gene co-occurrence. Others: 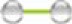 represents textmining; 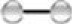 represents co-expression; 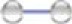 represents protein homology.

Kyoto Encyclopedia of Genes and Genomes (KEGG) online service tools KAAS were used to annotate protein's KEGG database description. Then the annotations were mapped on KEGG pathway database using KEGG mapper. KEGG enrichment analysis revealed the differentially expressed proteins were enriched in Glucagon signaling pathway, Inflammatory mediator regulation of TRP channels, Aldosterone synthesis and secretion, ECM-receptor interaction, cAMP signaling pathway, Oxidative phosphorylation, Ca^2+^ signaling pathway, MAPK signaling, and NF-κB signaling pathway (Fig. 5). The differentially expressed proteins were introduced into the web-tool STRING to generate protein-protein interaction networks (Fig. 6).

##### Verification of Differentially Expressed Proteins by Western Blot and Real-Time PCR

**Fig. 7.**
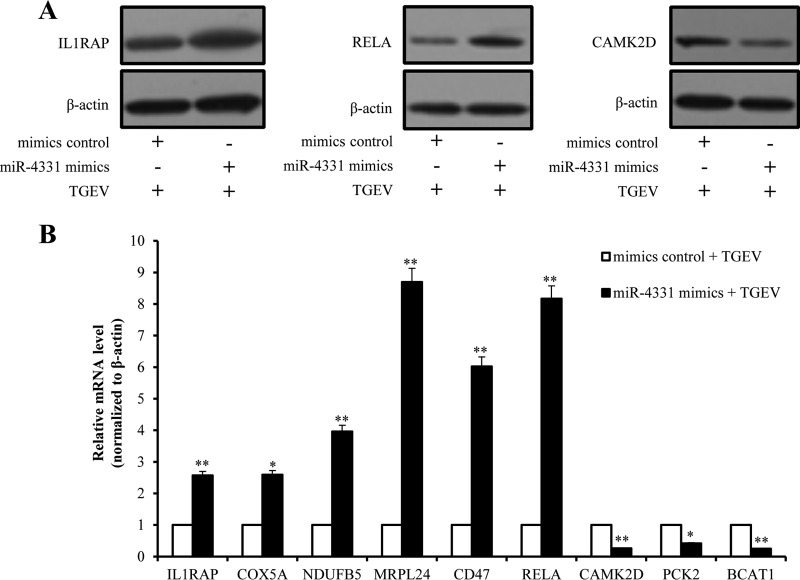
**Validation of differentially expressed proteins.** *A,* Western blot analysis of IL1RAP, RELA, and CAMK2K; *B,* Real-time PCR analysis of the differentially expressed proteins that are related to mitochondrial. Data are representative three independent experiments. * *p* < 0.05; ** *p* < 0.01.

The transcription and expression levels of some differentially expressed proteins were verified using Western blot and real-time PCR. The Western blot analysis revealed that the protein levels of IL1RAP and RELA in PK-15 cells were dramatically increased by miR-4331 overexpression (Fig. 7*A*). The mRNA levels of 9 mitochondria-related differentially expressed proteins, including IL1RAP, COX5A, NDUFB5, MARPL24, MARPL1, RELA, CAMKA2D, PCK2, and BCAT1, were confirmed by real-time PCR (Fig. 7*B*). The Western blot and real-time PCR results were consistent with the proteomic data.

##### IL1RAP Promotes TGEV-induced Mitochondrial Damage and Activates p38 MAPK Signaling

**Fig. 8.**
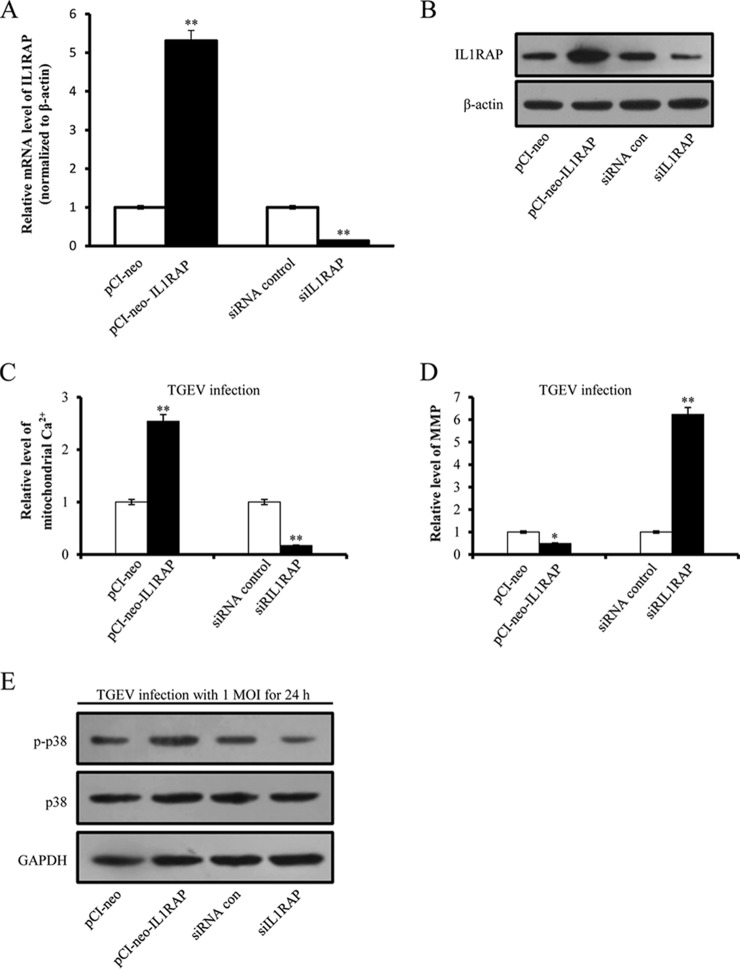
**Effects of IL1RAP on mitochondrial damage and p38 MAPK pathway.** *A,* The relative mRNA level of IL1RAP in PK-15 cells transfected with pCI-neo-IL1RAP and siIL1RAP; *B,* The effects of pCI-neo-IL1RAP and siIL1RAP on expression of IL1RAP; *C,* The effect of IL1RAP on mitochondrial Ca^2+^ level during TGEV infection; *D,* The effect of IL1RAP on MMP during TGEV infection; *E,* The effect of IL1RAP on p38 phosphorylation. Data are representative three independent experiments. * *p* < 0.05; ** *p* < 0.01.

According proteomic analysis, overexpression of miR-4331 may lead to an increase of IL1RAP protein level. It is reported that IL1RAP is a component of IL-1 protein complex (22) and IL-1 is a activator of p38 MAPK (23). We previously found that TGEV infection reduced MMP and activated p38 MAPK pathway (5). Therefore, we presume that IL1RAP might play a role in TGEV-induced decrease of MMP and activation of p38 MAPK. To explore the effects of IL1RAP on mitochondria and p38 MAPK pathway, we constructed pCI-neo-IL1RAP to overexpress IL1RAP and synthesized siRNA of IL1RAP, siIL1RAP, to silence IL1RAP. The results showed that pCI-neo-IL1RAP led to an increase IL1RAP at mRNA level (Fig. 8*A*) and protein level (Fig. 8*B*) and siIL1RAP reduced mRNA level (Fig. 8*A*) and protein level (Fig. 8*B*). Mitochondrial Ca^2+^ level was upregulated by pCI-neo-IL1RAP and downregulated by siIL1RAP (Fig. 8*C*). In addition, MMP level was suppressed by pCI-neo-IL1RAP and increased by siIL1RAP (Fig. 8*D*). As expected, phosphorylation level of p38 was enhanced by pCI-neo-IL1RAP and attenuated by siIL1RAP (Fig. 8*E*).

##### Activation of p38 MAPK Pathway Facilitates TGEV-induced Mitochondrial Damage

**Fig. 9.**
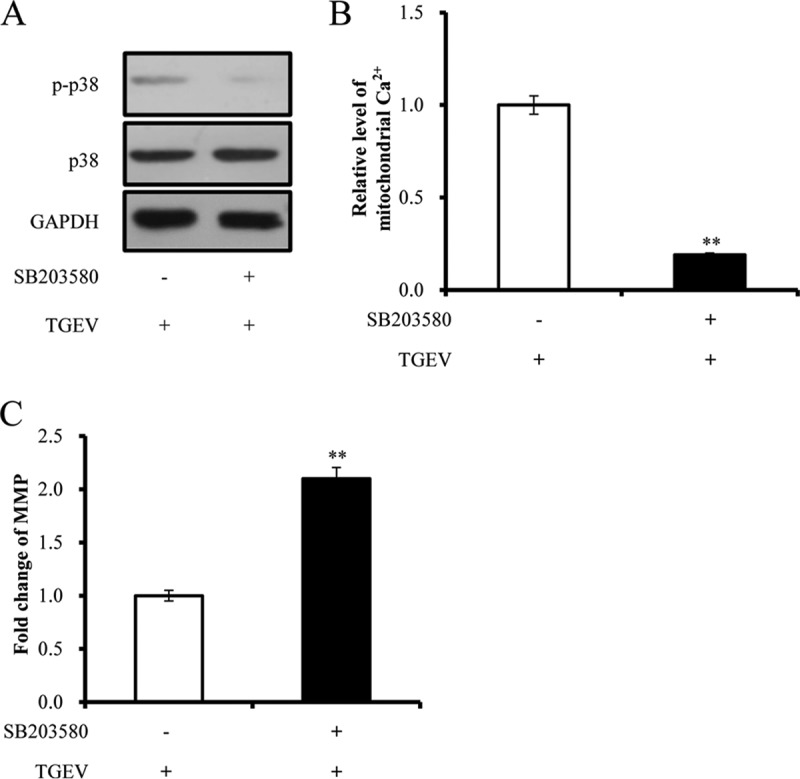
**Effects of p38 MAPK pathway on TGEV-induced mitochondrial damage.** *A,* Effect of p38 MAPK inhibitors SB203580 on p38 MAPK; *B,* Effect of p38 MAPK inhibitors on mitochondrial Ca^2+^ level during TGEV infection; *C,* Effect of p38 MAPK inhibitors on MMP during TGEV infection. Data are representative three independent experiments. ** *p* < 0.01.

To investigate whether the activation of p38 MAPK promotes TGEV-induced mitochondrial damage, PK-15 cells were treated with SB203580, the specific inhibitor of p38 MAPK, and infected with TGEV. As predicted, TGEV-induced phosphorylation of p38 was attenuated by SB203580 (Fig. 9*A*). Inhibition of p38 phosphorylation led to a decrease of mitochondrial Ca^2+^ level (Fig. 9*B*) and an increase of MMP during TGEV infection (Fig. 9*C*), indicating that p38 promotes TGEV-induced mitochondrial damage.

##### miR-4331 Promotes TGEV-induced Activation of p38 MAPK Pathway

**Fig. 10.**
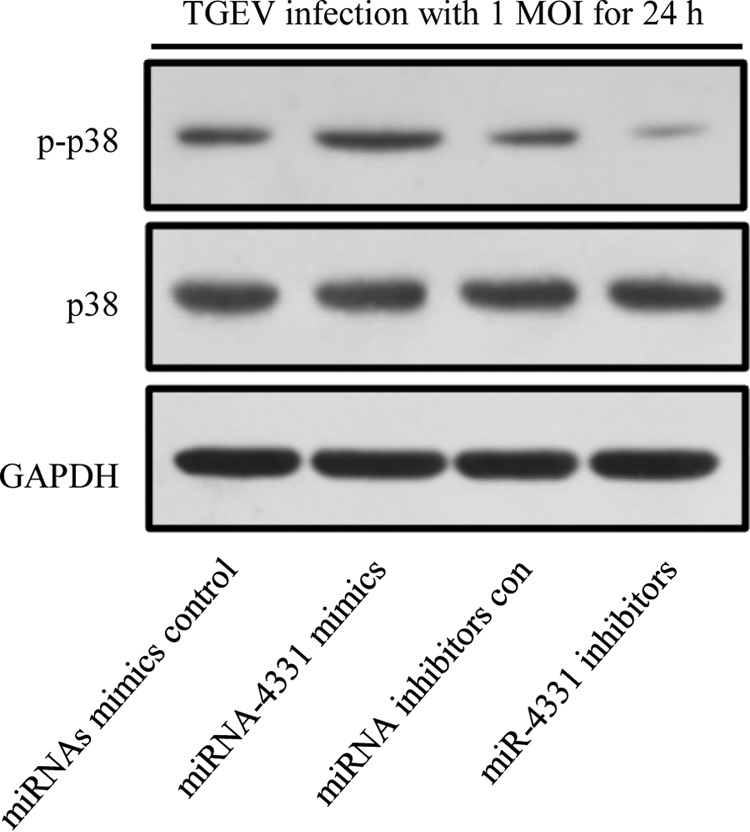
**Effect of miR-4331 on p38 MAPK pathway.**

We demonstrated that miR-4331 could upregulated IL1RAP expression via quantitative proteomic analysis and IL1RAP contributed to activation of p38 MAPK pathway via phosphorylating p38. However, whether miR-4331 can activate p38 pathway is unknown. To investigate the effect of miR-4331 on p38 MAPK, miR-4331 mimics or inhibitors was introduced into PK-15 cells. The phosphorylation level of p38 was detected. The result showed that TGEV-induced phosphorylation of p38 was enhanced by miR-4331 mimics and attenuated by miR-4331 inhibitors (Fig. 10), suggesting miR-4331 caused activation of p38 MAPK pathway.

##### RB1 is the Direct Target of miR-4331

**Fig. 11.**
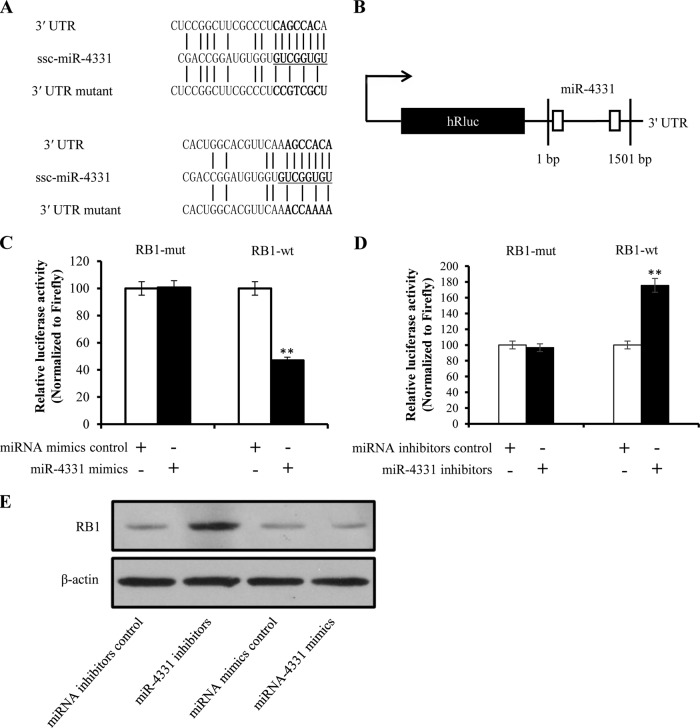
**Identification of miR-4331 targets.** *A,* Schematic overview of mutation of swine RB1 3′ UTRs sequence. The upper sequence is the binding site of miR-4331 in 3′ UTR of swine RB1. The middle is the sequence of mature miR-4331. The lower sequence is the mutated miR-4331 binding site in swine RB1. *B,* Schematic overview of construction of dual-luciferase report plasmid. The locations of the potential binding sites or their mutations are presented by blank boxes. *C,* The change of relative luciferase activities mediated by miR-4331 mimics. *D,* The change of relative luciferase activities mediated by miR-4331 inhibitors. *E,* Effect of miR-4331 on RB1 expression. Data are representative three independent experiments. ** *p* < 0.01.

To identify whether miR-4331 directly binds to the 3′ UTRs of miR-4331 targets, the miR-4331 binding sites of target mRNA were mutated with a 4-bp substitution (Fig. 11*A*). The wild type sequences of 3′ UTRs containing miR-4331 binding sites and mutated 3′ UTRs sequences of miR-4331 targets, in which miR-4331 seed sequence was mutated, were cloned into the 3′ UTR of Renilla luciferase in dual-luciferase reporter plasmid psiCHECK-2 to generate constructions, RB1-wt and RB1-mut (Fig. 11*B*). The constructs were co-transfected into PK-15 cells with either miR-4331 mimics/mimics control or miR-4331 inhibitors/inhibitors control. The Renilla luciferase activities of RB1-wt and RB1-mut were respectively reduced and improved by miR-4331 mimics (normalized to Firefly luciferase activity) (Fig. 11*C*). In contrast, the Renilla luciferase activities of RB1-wt and RB1-mut were respectively raised and decreased by miR-4331 inhibitors (normalized to Firefly luciferase activity) (Fig. 11*D*). The results revealed that miR-4331 directly binds to 3′ UTR of RB1 mRNA. To determine whether miR-4331 inhibits RB1 expression, either miR-4331 mimics or miRNA inhibitors control were transfected into PK-15 cells. RB1 protein level was tested using Western blot, showing that expression of RB1 was suppressed by miR-4331 mimics and improved by miR-4331 inhibitors (Fig. 11*E*).

##### RB1 Attenuates TGEV-induced Mitochondrial Ca^2+^ Level and Improves MMP During TGEV Infection

**Fig. 12.**
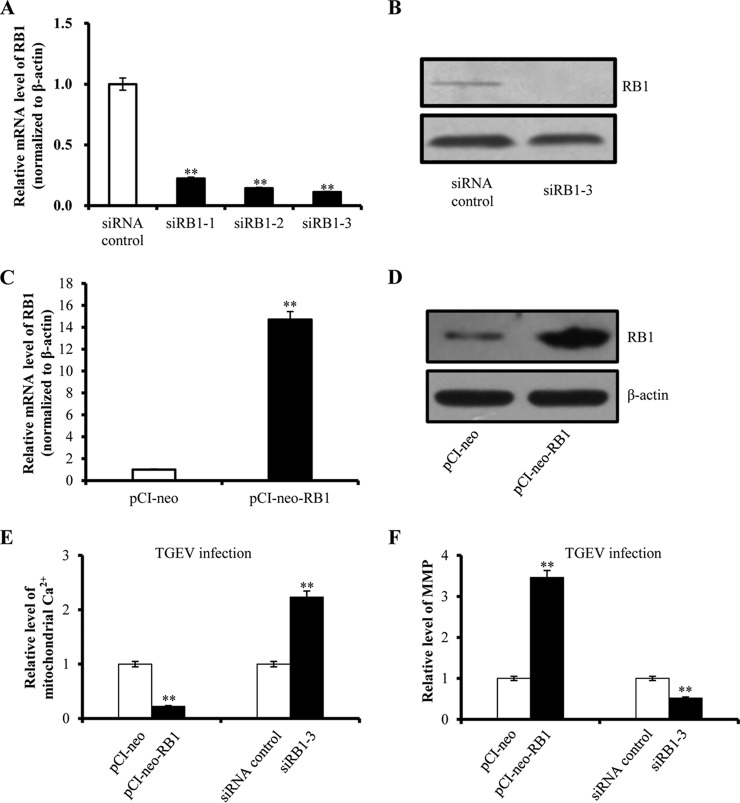
**Effect of RB1 on TGEV-induced mitochondrial damage.** *A* and *B,* Effect of RB1 siRNAs on RB1 transcription and expression; *C* and *D,* Effect of pCI-neo-RB1 on RB1 transcription and expression; *E,* Effect of RB1 on mitochondrial Ca^2+^ level during TGEV infection; *F,* Effect of RB1 on mitochondrial membrane potential level during TGEV infection. Data are representative three independent experiments. ** *p* < 0.01.

We demonstrated that miR-4331 facilitated mitochondrial damage and directly targeted RB1. However, whether miR-4331 affects mitochondria through targeting RB1 is unclear. To investigate the effect of RB1 on TGEV-induced mitochondrial damage, three siRNAs of RB1, siRB1–1, siRB1–2, and siRB1–3, were synthesized and respectively transfected into PK-15 cells to silence RB1. The silencing efficiency of RB1 siRNAs were evaluated, showing siRB1–3 was the most effective siRNA for RB1 silencing (Fig. 12*A* and 12*B*). Moreover, RB1 gene was cloned into eukaryotic expression plasmid pCI-neo, named pCI-neo-RB1, to overexpress RB1. pCI-neo-RB1 and pCI-neo were respectively transfected into PK-15 cells, followed by evaluation of mRNA and protein level. Expectedly, both the mRNA and protein level were upregulated (Fig. 12*C* and 12*D*). Then, PK-15 cells were transfected with either siRB1–3 or pCI-neo-RB1 and subsequently infected with TGEV for 24 h. The mitochondrial Ca^2+^ level and MMP were measured. The results showed that mitochondrial Ca^2+^ level was decreased by pCI-neo-RB1 and increased by siRB1–3 (Fig. 12*E*). In addition, MMP was upregulated by pCI-neo-RB1 and decreased by siRB1–3 (Fig. 12*F*). The results reveal that miR-4331 promotes TGEV-induced mitochondrial damage via targeting RB1.

##### RB1 Suppresses IL1RAP Expression and p38 MAPK Signaling

**Fig. 13.**
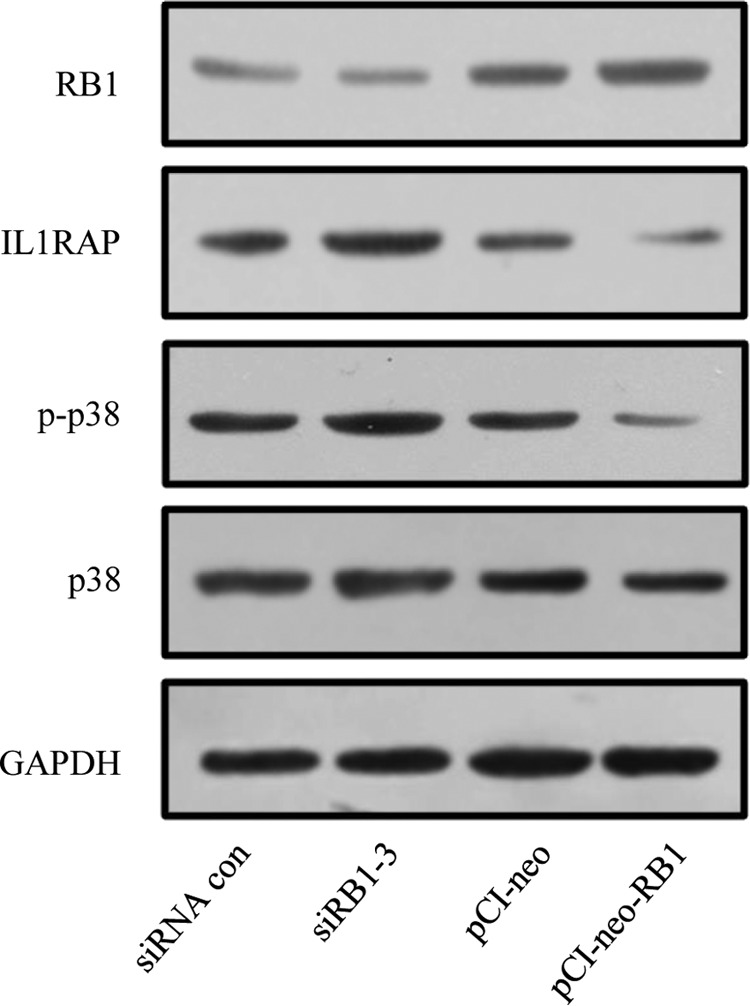
**Effect of RB1 on IL1RAP and activation of p38 MAPK.**

We showed that miR-4331 could aggravate TGEV-induced mitochondrial damage via targeting RB1 and activating p38 MAPK pathway. Therefore, we presumed that miR-4331 is likely to regulate IL1RAP and p38 MAPK signaling through its target RB1. We silenced RB1 using siRB1–3 and overexpressed RB1 using pCI-neo-RB1. IL1RAP, p38, and p-p38 were analyzed by Western blotting. It was found that RB1 was suppressed by siRB1–3 (Fig 13). Silencing RB1 caused an upregulation of IL1RAP and p-p38 (Fig. 13). Reversely, overexpression of RB1 led to a suppress of IL1RAP and p-p38 (Fig. 13).

## DISCUSSION

In this study, we investigated the effects of miR-4331 on mitochondrial damage induced by TGEV infection via targeting RB1. The results showed that miR-4331 promoted TGEV-induced mitochondrial damage via targeting RB1, upregulating IL1RAP, and enhancing TGEV-induced activation of p38 pathway. Our findings reveal a novel regulatory effect of miR-4331 on TGEV-induced mitochondrial damage.

It is reported that TGEV infection caused the alterations of proteomes in PK-15 cells, including upregulated STAT1 and many ISGs (ISG15, IFIT1, IFIT2, IFIT3, IFIT5, OAS1, OAS2, and Mx1) (24). In this study, Mx1 was differentially decreased by miR-4331 in contrast to upregulation in TGEV-infected PK-15 cells. It indicates that miR-4331 plays a negative role in TGEV-induced Mx1 expression. In addition, although STAT1 and many ISGs, including ISG15, IFIT1, IFIT2, IFIT3, OAS1, OAS2, Mx1, were detected using TMT approach, only Mx1 was altered by miR-4331 in TGEV-infected PK-15 cells. The likely reason is that miR-4331 downregulated TGEV-induced STAT1 and these ISGs, including ISG15, IFIT1, IFIT2, IFIT3, OAS1, OAS2, to normal level. In contrast to upregulation in TGEV-infected PK-15 cells, Mx1 was differentially decreased by miR-4331 in TGEV-infected PK-15 cells. It indicates that miR-4331 plays a negative role in TGEV-induced Mx1 expression.

In this study, RB1 is identified as the target of miR-4331 and affects mitochondrial function. RB1 is a suppressor of cell death via binding and repressing transcription factor E2Fs. The hypophosphorylated RB1, the active form of RB1, binds to and sequesters transcription factor E2F1 to operate as an inhibitor of E2Fs (25). C-terminal region of RB1 has a E2F1-specific binding site that is sufficient to repress E2F1-induced apoptosis, so the C-terminal interaction site on RB1 acts as a potent inhibitor of E2F1 (26). A pool of evidence shows RB1 localizes in mitochondria (27). Therefore, a set of reports highlights a series of links between RB1 and mitochondrial function. RB1 promotes mitochondrial biogenesis for erythropoiesis (28). Loss of RB1 caused a decrease of mitochondrial mass, downregulated mitochondrial function, oxidative phosphorylation, MMP, and accumulation of hypopolarized mitochondria (29), implying that suppression of RB1 leads to mitochondrial damage. These results further support our conclusion that RB1 weakens TGEV-induced mitochondrial damage. Here, RB1 was identified as the target of miR-4331 and inhibited by miR-4331. Whereas, RB1 was not identified as the differentially expressed protein using TMT, it may be because of low expression level of RB1 and the insufficient sensitivity of this technology.

Cytokines of the IL-1 family are considered critical regulators of intestinal mucositis (30). IL-1 is a key proinflammatory cytokine that can initiate a series of signaling resulting in activation of NF-κB signaling pathway. IL-1 interacts with L1RAP and IL-1 receptor type I (IL-1RI) to form IL-1 complex (31), which mediates the activation of p38 MAPK signaling in presence of IL-1β treatment (32). It is in consistent with our finding that IL1RAP can lead to the activation of p38 MAPK pathway. Moreover, we found miR-4331 caused an upregulation of RELA, which is the p65 subunit of NF-κB. NF-κB is a key activator of inflammation and is activated during TGEV infection (33, 34). Therefore, we speculate miR-4331 may function as a regulator of TGEV-induced NF-κB pathway through regulating IL1RAP and RELA.

## DATA AVAILABILITY

The mass spectrometry proteomics data have been deposited to the ProteomeXchange Consortium via the PRIDE partner repository with the data set identifier PXD008174 (http://www.ebi.ac.uk/pride/archive/) (18).

## Supplementary Material

Supplemental Data

## Footnotes

^1^ The abbreviations used are:

TGEV: transmissible gastroenteritis virus
PK-15 cells: porcine kidney (PK) 15 cells
miRNA: microRNA
MMP: mitochondrial membrane potential
UTR: untranslated region
GO: gene ontology
KEGG: kyoto encyclopedia of genes and genomes
MOI: multiplicity of infection
TMT: tandem mass tags
hpi: hours post-infection
hpt: hours post-transfection
HPLC: high performance liquid chromatography
MS: mass spectrometry.
