# Supplementary material for: microRNA-4331 Promotes Transmissible Gastroenteritis Virus (TGEV)-induced Mitochondrial Damage Via Targeting RB1, Upregulating Interleukin-1 Receptor Accessory Protein (IL1RAP), and Activating p38 MAPK Pathway In Vitro
Source: Mol Cell Proteomics. 2017 Dec 7;17(2):190–204. doi: 10.1074/mcp.RA117.000432 (PMC5795386; doi:10.1074/mcp.RA117.000432)
Supplement: Supplemental Data [file supp_17_2_190__index.html]

microRNA-4331 promotes TGEV-induced mitochondrial damage via targeting RB1, up-regulating IL1RAP, and activating p38 MAPK pathway in vitro — miR-4331 promotes mitochondrial damage via p38 MAPK pathway — microRNA-4331 Promotes Transmissible Gastroenteritis Virus (TGEV)-induced Mitochondrial Damage Via Targeting RB1, Upregulating Interleukin-1 Receptor Accessory Protein (IL1RAP), and Activating p38 MAPK Pathway In Vitro — miR-4331 Promotes Mitochondrial Damage Via p38 MAPK Pathway — Supplemental Data 

# microRNA-4331 Promotes Transmissible Gastroenteritis Virus (TGEV)-induced Mitochondrial Damage Via Targeting RB1, Upregulating Interleukin-1 Receptor Accessory Protein (IL1RAP), and Activating p38 MAPK Pathway *In Vitro*

## Supplemental Data

- Sequences of primers and siRNAs - Sequences of primers and siRNAs
- Predicted targets of miR-4331 - Predicted targets of miR-4331.
- MS identified information - MS identified information
- Differentially expressed proteins - Information of defferentially expressed proteins
